# Somatostatinoma Presented as Double-Duct Sign

**DOI:** 10.1155/2019/9506405

**Published:** 2019-05-09

**Authors:** Ali Zakaria, Nour Hammad, Cynthia Vakhariya, Michael Raphael

**Affiliations:** ^1^Division of Gastroenterology, Providence-Providence Park Hospital and Medical Center, Michigan State University/College of Human Medicine, Southfield, Michigan, USA; ^2^Department of Internal Medicine, Providence-Providence Park Hospital and Medical Center, Michigan State University/College of Human Medicine, Southfield, Michigan, USA; ^3^Division of Hematology & Oncology, Providence-Providence Park Hospital and Medical Center, Michigan State University/College of Human Medicine, Southfield, Michigan, USA

## Abstract

Somatostatinoma is a rare neuroendocrine tumor with an incidence rate of 1 in 40 million people. It presents mostly as asymptomatic tumor diagnosed incidentally on imaging or surgery when evaluating or treating possible causes of abdominal pain. It also can present with vague symptoms, or as a clinical triad of glucose intolerance, steatorrhea, and achlorhydria. The majority of somatostatinomas are present in the pancreatic head, followed by the duodenum, the pancreatic tail, and rarely the ampulla of Vater. The prognosis is poor as more than 77% of cases present as advanced disease with local invasion or distant metastasis. Surgical resection is the main treatment for early stage disease. Other treatment options include somatostatin analogue, molecular targeted therapy, and cytotoxic chemotherapy. The scarcity of somatostatinoma cases led to the lack of fully formulated treatment options. Herein, we present a 43-year old male patient who was referred by his primary care physician to our gastroenterology clinic due to elevated liver function test and double-duct sign on CT scan. We performed an ERCP, which revealed 2 cm ampullary lesion with upstream obstruction. Biopsies were taken and histopathology was unrevealing. He underwent a laparoscopic pancreaticoduodenectomy with histopathology revealed stage IIb somatostatinoma. Treating physicians should hold a high index of suspicion and maintain a broad differential diagnosis of elevated liver enzymes.

## 1. Introduction

Somatostatinoma is a rare neuroendocrine tumor with an incidence rate of 1 in 40 million people. It presents mostly as asymptomatic tumor diagnosed incidentally on imaging or surgery when evaluating or treating possible causes of abdominal pain. It can also present with vague symptoms, or as a clinical triad of glucose intolerance, steatorrhea, and achlorhydria. Somatostatinoma mostly involves the pancreatic head followed by the duodenum.

Due to vague symptomatology it presents in >77% of cases as advanced disease with local invasion or distant metastasis. The rarity of the disease led to lack of fully formulated treatment protocol, with surgical resection, somatostatin analogue, molecular targeted therapy, and cytotoxic chemotherapy being available options. The prognosis is poor in advanced diseases.

## 2. Case Report

This is a 43-year-old white male patient with past medical history of hypertension and obesity. He was referred to the gastroenterology clinic from his primary care physician due to elevated liver function test found on routine annual physical exam. At the time of evaluation he had no complaint, and he denied any abdominal pain, nausea, vomiting, jaundice, weight changes, skin lesions, or discoloration. He also denied any prior history of illicit drug use, high risk sexual behavior, sexually transmitted diseases, or family history of liver or autoimmune diseases. His physical examination did not reveal any skin lesions suspicious for neurofibromatosis-1.

His initial workup revealed a random glucose level of 85 mg/dl, calcium level 9.5 mg/dl, total bilirubin 1.3 mg/dl, direct bilirubin 0.6 mg/dl, alkaline phosphatase 640 U/L, AST 220 U/L, ALT of 494 U/L, total protein 7.1 g/dl and albumin 4.6 g/dl. Further workup revealed negative viral hepatitis panel, antinuclear antibodies, anti-mitochondrial antibodies and anti-smooth muscle antibodies. Iron studies, ceruloplasmin and alpha-1 antitrypsin were within normal limits.

**Figure 1:**
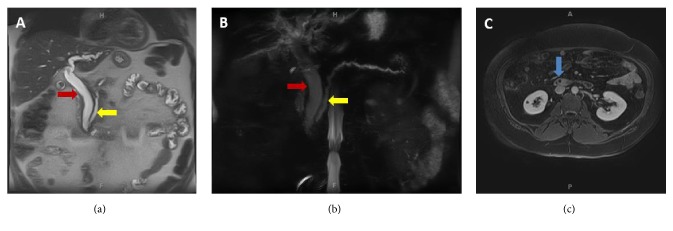
MRI abdomen with and without contrast: (a) T2 image and (b) MRCP image reveal moderate to severe intrahepatic biliary ductal dilation. The common duct measures 1.5 cm in greatest diameter “red arrow”. The pancreatic duct is also dilated measuring up to 0.6 cm at the head “yellow arrow”. No intraluminal filling defect. (c) T1 image with contrast reveals mild distal common bile duct wall enhancement “blue arrow”.

Abdominal ultrasonography revealed normal liver size and echotexture with no lesions, intrahepatic biliary duct dilatation, common bile duct dilatation 18 mm, contracted gallbladder with no cholelithiasis and no discrete pancreatic mass or ductal dilatation were identified. Magnetic resonance image/magnetic resonance cholangiopancreatography (MRI/MRCP) was performed and revealed moderate to severe intrahepatic and extra hepatic biliary ductal dilatation with mild enhancement of the distal common bile duct without choledocholithiasis or discrete mass, and pancreatic duct dilation with normal pancreas [Figure 1].

**Figure 2:**
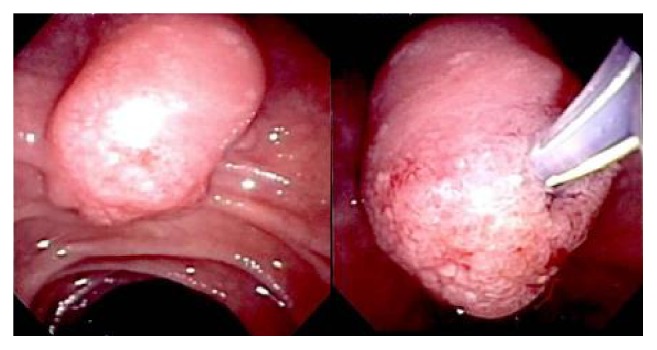
Endoscopic (ERCP) images reveal a 2cm ampullary mass.

An endoscopic retrograde cholangiopancreatography (ERCP) was performed and revealed a large ampullary mass distorting the anatomy, which precluded cannulation of the common bile duct [Figure 2]. During his ongoing evaluation the patient developed pruritus and his repeated liver function test revealed stable levels with numbers around his initial presentation. An advanced endoscopist repeated the ERCP, which revealed 2 cm ampullary lesion with upstream obstruction, pancreatic duct dilatation measuring 4.5 mm, intra- and extra-hepatic biliary dilatation with common bile duct measured 16 mm. A biliary sphincterotomy and balloon sweep was performed, and biopsies were taken of the ampullary tissue. Histopathology revealed congested mixed acute and chronic inflammation with reactive epithelial changes, negative for malignancy. The CA 19-9 level was elevated at 51.4 units/mL.

**Figure 3:**
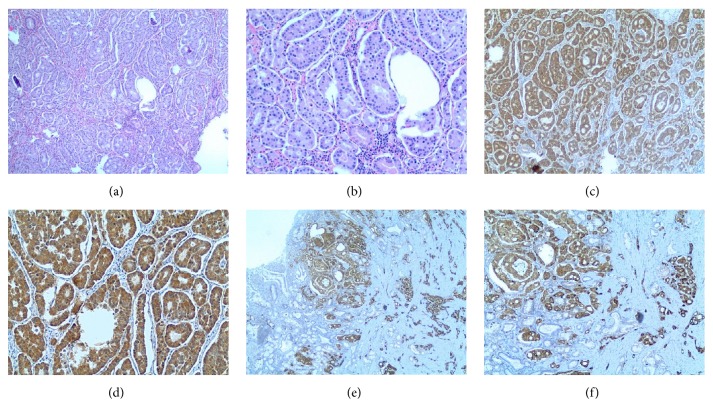
Histopathology images H&E [4x (a) and 0x (b)] reveal ampullary neuroendocrine tumor infiltrating the mucosa, submucosa, and muscularis propria with angiolymphatic invasion. Immunohistochemical stain images are positive for somatostatin [4x (c) and 10x (d)], and synaptophysin [2x (e) and 4x (f)].

The patient underwent laparoscopic pancreaticoduodenectomy (Whipple's procedure) with regional lymphadenectomy. Histopathology was consistent with 1.3 cm ampullary neuroendocrine tumor infiltrating the mucosa, submucosa and muscularis propria with angiolymphatic invasion. Immunohistochemical stains were positive for somatostatin, and negative for gastrin, serotonin and pancreatic polypeptide [Figure 3]. There was no local invasion, the specimen margins were negative, and 1/18 lymph nodes were positive. The patient was diagnosed with stage IIb (T2 N1 M0) somatostatinoma.

The post resection surveillance at 3, 6, and 12 months revealed a normal history and physical examination, a fasting somatostatin level of 41, 28, and 19 pg/ml, respectively, and a CT scan abdomen and pelvis with no evidence of recurrence. He is still under yearly surveillance with normal history, physical examination and fasting somatostatin level for the last four years.

## 3. Discussion

D or delta cells are neuroendocrine cells located in the gastrointestinal (GI) tract and pancreatic islets of Langerhans that secret somatostatin [1]. Somatostatin is a cyclic tetradecapeptide with major inhibitory action on the GI tract hormonal secretion, namely, gastric acid, pepsin and enterochromaffin-like (ECL) cell histamine. Neurotransmitters including acetylcholine and GI peptides are furthermore inhibited by somatostatin. Moreover, somatostatin enhances early gastric emptying and distal intestinal motility and delays late phase gastric emptying and gastroduodenal motility. It also hinders intestinal nutrient absorption and splanchnic blood flow [1].

Somatostatin was initially analyzed from the hypothalamus of sheep in 1973 and was later found to be present in the pancreas, lung, adrenal and thymic tissue [1, 2]. Somatostatinoma is a rare tumor with an incidence rate of 1 in 40 million people and an average age of incidence of 50 in men and 52 years in women [2, 3]. Somatostatinoma occurs in association with multiple neuroendocrine syndrome type 1 (MEN-1) in 45% of cases; it is considered one of the least common functioning enteropancreatic neuroendocrine tumors occurring in <1% of MEN-1 cases [4]. It develops also in 10% of patients with neurofibromatosis type 1 [5]. Patients diagnosed with somatostatinoma should be screened for other components of MEN-1 by either serum calcium level (parathyroid hormone and ionized calcium level can be added to enhance sensitivity and specificity) or DNA-based genetic testing. A thorough skin exam looking for signs of NF-1 should also be considered. Larson L et al. initially described somatostatinoma in 1977. They found the tumor incidentally in the pancreas during cholecystectomy. They later performed a retrospective analysis of tumor records and documented another case of pancreatic D cell tumor. They associated the classic triad of glucose intolerance, steatorrhea, and achlorhydria in both patients with somatostatinoma in accordance with somatostatin physiologic effects [6]. It was noted that the somatostatinoma syndrome triad was mostly seen in pancreatic tumors while obstructive symptoms were associated with duodenal masses [7].

The size and location of the tumor has a major influence on the severity and nature of symptoms [8]. The majority of somatostatinomas are present in the pancreatic head (45%), followed by the duodenum (19%), the pancreatic tail (13%) and later the ampulla of Vater (6%) [2]. If symptomatic, somatostatinoma patient's most common complaints are weight loss and abdominal pain [2]. Duodenal somatostatinomas were associated with jaundice and cholestasis in addition to the abdominal pain and weight loss. Many of the somatostatinomas are silent or present with vague symptoms making the diagnosis of such tumors an enigma. Many reported tumors were found incidentally on imaging or surgery when evaluating or treating possible causes of abdominal pain and diagnosis was confirmed by histopathology after resection [7]. When suspected due to presence of somatostatinoma triad, a fasting plasma somatostatin level of more than 30 pg/ml is diagnostic. Other modalities that may aid in diagnosis include computed tomography (CT), magnetic resonance imaging (MRI), endoscopic ultrasound (EUS), somatostatin receptor scintigraphy (OctreoScan) and function positron emission tomography (PET). OctreoScan uses radiolabeled octreotide ([111-In] pentetreotide); it has the advantage of instantaneous whole body scanning, which allows extra-abdominal metastasis detection. Guidelines have different recommendations on when to obtain this study, but experts suggested using this technique as adjunct for tumor staging if finding would change patient management [2]. The functional PET technique uses different tracers (e.g., 68-Ga-DOTATOC, or the newly FDA approved 68-Ga-DOTATATE). It provides higher resolutions, which improves sensitivity for small lesions detections.

Somatostatinoma D cells are located in the submucosa and therefore the yield of the endoscopic biopsies is low. Only 60 to 83% of the biopsies have a positive yield for diagnosis [9]. Histopathological presentation of D cell is marked by the presence of large secretory granules of medium to low intensity. Pathologists use immuno-reactive somatostatin to confirm the presence of the targeted somatostatin in the granules. The spherical, laminated, and mineralized concretions, known as psammoma bodies, are pronounced histological findings that are seen in duodenal rather than pancreatic somatostatinomas [10].

Due to the ambiguity of symptoms at presentation of the somatostatinoma, many of the tumors are diagnosed late. The vast majority of the cases (77%) were diagnosed after distant metastases or local invasion, with the liver being the most common site of invasion (42%). Other areas of tumor invasion are accounted by the lymph nodes (39%), duodenum (13%), spleen (6%), common bile duct, colon, stomach, left kidney, bone and pancreas [2].

The scarcity of somatostatinoma cases led to the lack of fully formulated treatment options. Surgery is still the preferred treatment for smaller tumors of less than 2x2 cm; however, 70-92% of cases present with advanced disease for which surgery is not an option [11]. Larger tumors, locally infiltrative, or tumors with lymph node metastases will mandate pancreaticoduodenectomy. Tumors with extensive distant metastases that are challenging to resect will require other treatment options: Debulking, somatostatin analogue (e.g., octreotide, lanreotide), molecular targeted therapy (e.g., everolimus, sunitinib), and cytotoxic chemotherapy [12, 13]. 5-Flourouracil and streptozocin are chemotherapy agents that were incorporated in the treatment of previous somatostatinoma patients with limited benefit [2]. In patients with extensive liver metastasis other treatment modalities that can be used include hepatic artery embolization, chemoembolization and if small lesions (<3cm) radiofrequency ablation and cryoablation [14]. Liver transplantation is still an investigational approach with no enough data to provide any recommendation for this modality as a cure. Resection of localized somatostatinoma is definitive; however, the prognosis of patient diagnosed with metastatic disease is poor in general with reported survival period of one to two years [8, 15]. Due to limited data regarding somatostatinoma, the survival rate estimate can be roughly reflected from the five- and ten-year survival of the patient who underwent resection of pancreatic neuroendocrine tumors, which was estimated to be 64-75%, and 45-71% in stage I-II disease, compared to 19-60%, and 8-33% in stage III-IV disease, respectively [16]. The National Comprehensive Cancer Network recommendation regarding post-treatment surveillance of somatostatinoma consists of (a) History and physical examination, fasting somatostatin level and imaging studies with CT scan or MRI after 3-12 months; (b) History and physical exam, fasting somatostatin level (every 6-12 months) and imaging studies as clinically indicated after 12 months up until 10 years [17].

## 4. Conclusion

Somatostatinoma is a very rare neuroendocrine tumor. We reported this case to emphasize the importance of holding a high index of suspicion and maintaining a broad differential diagnosis of elevated liver enzymes. Unfortunately, there is no standardized treatment regimen so far, and the overall survival is poor due to the advanced stage on presentation.
